# Ion-Channel-Targeting Scorpion Recombinant Toxin as Novel Therapeutic Agent for Breast Cancer

**DOI:** 10.3390/toxins17040166

**Published:** 2025-03-26

**Authors:** Natalia Mata de los Rios, Argel Gastelum-Arellanez, Herlinda Clement, Karely Álvarez-Cruz, Diana Romero-Terrazas, Carolina Alvarado-González, Luis Carlos Hinojos-Gallardo, Gerardo Corzo, Gerardo Pável Espino-Solis

**Affiliations:** 1 Traslational Research Laboratory, Facultad de Medicina y Ciencias Biomédicas, Autonomous University of Chihuahua, Circuito Universitario s/n, Campus II, Chihuahua 31125, Mexico; p318968@uach.mx (N.M.d.l.R.); a351874@uach.mx (K.Á.-C.);; 2 Facultad de Medicina y Ciencias Biomédicas, Autonomous University of Chihuahua, Circuito Universitario s/n, Campus II, Chihuahua 31125, Mexico; argastelum@uach.mx (A.G.-A.);; 3 Instituto de Biotecnología—UNAM, Universidad Nacional Autónoma de México, Av. Universidad 2001, Col. Chamilpa, Cuernavaca 62210, Mexicogerardo.corzo@ibt.unam.mx (G.C.); 4 Laboratorio Nacional de Citometría de Flujo, Facultad de Medicina y Ciencias Biomédicas, Autonomous University of Chihuahua, Circuito Universitario s/n, Campus II, Chihuahua 31125, Mexico

**Keywords:** ion channel, cell viability, proliferation, breast cancer, *C. coahuliae*, antineoplastic, doxorubicin

## Abstract

Breast cancer remains the leading cause of cancer-related mortality among women worldwide, with limited therapeutic efficacy due to treatment resistance and adverse effects. Emerging evidence suggests that ion channels play crucial roles in tumor progression, regulating proliferation, apoptosis, migration, and metastasis. Voltage-gated potassium (Kv) and sodium (Nav) channels have been implicated in oncogenic signaling pathways. Scorpion venom peptides, known for their selective ion-channel-blocking properties, have demonstrated promising antineoplastic activity. This study explores the potential therapeutic applications of bioactive fractions derived from *Chihuahuanus coahuilae*, in breast cancer cell lines. Through chromatographic separation, mass spectrometry, and functional assays, we assess their effects on cell viability, proliferation, and ion channel modulation. Our preliminary data suggest that these venom-derived peptides interfere with cancer cell homeostasis by altering ion fluxes, promoting apoptosis, and inhibiting metastatic traits. These findings support the therapeutic potential of ion-channel-targeting peptides as selective anticancer agents. Further investigations into their molecular mechanisms may pave the way for novel, targeted therapies with improved efficacy and specificity for breast cancer treatment.

## 1. Introduction

Cancer encompasses a group of diseases characterized by abnormal cells that proliferate uncontrollably, invade adjacent tissues, and metastasize to distant sites [1]. Similarly, breast cancer comprises a heterogeneous group of malignancies classified into three main subtypes based on their molecular and histological profiles: those expressing estrogen and/or progesterone hormone receptors, those expressing the HER2 receptor, and triple-negative breast cancers that lack expression of both hormone receptors and HER2 [2]. Treatment approaches are tailored according to their molecular profile and typically involve surgical intervention, chemotherapy (e.g., doxorubicin), radiotherapy, hormonal therapy, and targeted therapy [3]. However, in Chihuahua, Mexico, most cases are diagnosed at advanced stages, resulting in a low five-year survival rate. Consequently, breast cancer ranks as the leading cause of cancer-related mortality, with a rate of 17.9 per 100,000 women, and exhibits the highest incidence at 28.2% [4]. Furthermore, the limited specificity of current treatments leads to significant adverse effects, while developing resistance mechanisms underscores the urgent need for novel therapeutic strategies. This has driven extensive research into cancer molecular biology and the identification of novel compounds with antineoplastic properties.

During carcinogenesis, tumor cells acquire distinct characteristics that enable malignant transformation, commonly referred to as the hallmarks of cancer. These include sustained proliferative signaling, evasion of growth suppressors and apoptosis, and tissue invasion leading to metastasis [5]. While these features largely arise from mutations in oncogenes and tumor suppressor genes, growing evidence suggests that ion channel dysregulation also plays a significant role in cancer progression. The overexpression or aberrant function of ion channels, particularly voltage-gated potassium (Kv) and sodium (Nav) channels has been implicated in cellular proliferation, apoptosis resistance, migration, invasion, and metastasis [6].

The major potassium channels associated with cancer include Kv1.3, Kv10.1, and Kv11.1, which have been linked to proliferative signaling and apoptosis resistance. Specifically, Kv1.3 overexpression in the plasma membrane regulates cell proliferation by inducing hyperpolarization during the S phase of the cell cycle. This increases the electrochemical gradient for calcium influx, which in turn activates potassium and chloride channels, leading to osmotic cell contraction, a prerequisite for mitotic progression [6]. Notably, tumor cells often downregulate proapoptotic factors such as Bax, which acts as an inhibitor of these channels, thereby enhancing apoptosis resistance [6].

In contrast, the role of sodium channels in cancer remains less well understood. However, Nav channel overexpression has been associated with more aggressive phenotypes characterized by enhanced invasion, migration, and metastatic potential. Pharmacological inhibition of Nav1.5, Nav1.6, and Nav1.7 suppresses migration and invasion in experimental models, although the precise underlying mechanisms remain incompletely understood [6].

Given these findings, ion channels have emerged as promising therapeutic targets. Inhibiting specific channels expressed in the plasma and mitochondrial membrane could exert antiproliferative and proapoptotic effects, with the advantage of selectively targeting neoplastic cells [7]. This has led to increasing interest in the study of ion channel blockers, including bioactive compounds derived from animal venoms.

Scorpion venom has attracted significant attention due to its potent neurotoxic, paralytic, and cytotoxic properties. In humans, scorpion venom can induce severe pain, autonomic dysfunction, and even fatal systemic complications. Biochemical characterization has revealed that scorpion venom is a complex mixture of amines, enzymes, salts, nucleotides, amino acids, mucoproteins, lipids, peptides, and other unidentified bioactive substances [7,8]. The peptide components of scorpion venom are classified into disulfide-bridged peptides (DBPs) and non-disulfide-bridged peptides (NDBPs). DBPs are further categorized into long-chain peptides (50–80 amino acids), which primarily target sodium channels, and short-chain peptides (20–50 amino acids), which modulate potassium channels. The α-DBPs bind to site 3 of the Nav channel, delaying inactivation and thereby increasing neuronal excitability, whereas β-DBPs bind to site 4, shifting the voltage threshold for activation to more negative potentials, ultimately inducing inactivation. Short-chain peptides interact with potassium channels either by obstructing the central pore or modifying channel kinetics, thereby reducing conductance. Additionally, some peptides interact with chloride channels, leading to internalization and diminished ion flux, while others, such as calcins, function as ryanodine receptor (RyR) agonists, elevating intracellular calcium levels and inducing contractile paralysis [9]. Notably, scorpion venom peptides exhibit diverse pharmacological properties, including antibacterial, immunosuppressive, insecticidal, analgesic, and antineoplastic activities [9,10].

The anticancer potential of scorpion venom components has been extensively investigated. For instance, chlorotoxin, derived from *Leiurus quinquestriatus*, inhibits chloride channels, thereby reducing proliferation and migration in glioma, breast, and pancreatic cancer cells while enhancing the efficacy of cisplatin and doxorubicin. Similarly, Bm-12 from *Mesobuthus martensii Karsch* exerts comparable effects in glioma cells. Iberiotoxin, isolated from *Mesobuthus tumulus*, blocks potassium channels, suppressing proliferation, inducing S-phase cell cycle arrest, and triggering apoptosis in glioma and astrocytoma cells. Other scorpion-derived peptides, such as caribdotoxin, margatoxin, bengalin, neopladins, maurocalcins, and leptulipin, have demonstrated significant antineoplastic activities across various cancer models, including breast, lung, melanoma, and leukemia [7,11,12,13].

Beyond specific peptides, crude scorpion venoms have also exhibited promising anticancer properties. For instance, venom from *Hottentotta schach* reduced cell viability and induced apoptosis in MCF-7 breast cancer cells, while *Odontobuthus doriae* venom triggered necrosis and apoptosis through ROS-mediated mechanisms [14,15]. Similar effects were observed with *Buthus martensii Karsch* and *Rhopalurus junceus* venoms in breast cancer [16,17]. Recent studies by Alvarado-González et al. [10] employed reverse-phase liquid chromatography (HPLC-RP) to fractionate venom from *Chihuahuanus coahuilae*, identifying multiple fractions with potential antineoplastic activity. Thus, the present study aims to investigate whether recombinant fractions derived from *Chihuahuanus coahuilae* exhibit antineoplastic activity in breast cancer cell lines and whether these compounds may contribute to the development of more effective and targeted therapies for breast cancer.

## 2. Results

### 2.1. Purification and Molecular Characterization of Fraction 43 from Chihuahuanus coahuilae Scorpion Venom

The total venom of *Chihuahuanus coahuilae* was fractionated by reverse-phase high-performance liquid chromatography (RP-HPLC), following the methodology of Alvarado-González et al. [10] (Supplementary Materials, Figures S1 and S2). This approach led to the identification of Fraction 43 (rChcoh-43), a novel component characterized by its toxicity in mice with 100% mortality, when 2 μg in male mice (CD-1, 20 g body weight) by intracranial (ic) injection were tested (additional information on the toxicity of the recombinant rChcoh-43 toxin is provided in the Supplementary Materials, Table S1). The purified fraction underwent N-terminal sequencing via Edman degradation, revealing a partial sequence of 13 amino acid residues. Using this sequence and the molecular mass of Chcoh-43 (6736.24 Da), a database search (https://www.uniprot.org/blast, accessed on 28 November 2024) was performed to identify homologous sequences with high identity. The retrieved sequences were aligned using multiple sequence alignment (MSA), and gene-specific oligonucleotides were designed to amplify and characterize the full-length gene from cDNA.

### 2.2. N-Terminal Sequencing and 3′ RACE

Toxins from *Chihuahuanus coahuilae* were purified and analyzed as outlined in Section 1 for N-terminal sequencing. The N-terminal region of the Chcoh-43 toxin (*Ch. coahuilae*) was partially sequenced, revealing a 15-amino acid sequence (KKDGYPVTKYGQVFN). This sequence was utilized to design primers for subsequent 3′ RACE assays. Venom glands from *Ch. coahuilae* specimens served as the source for RNA extraction. Only 3′ RACE-amplified transcripts (Section 2) that exhibited the expected size on agarose gels were selected for purification and sequencing. The deduced amino acid sequences from these transcripts are presented in Table 1. The translation of transcripts to protein sequences was conducted using the Expasy Translate tool (https://web.expasy.org/translate/, accessed on 15 December 2024).

To classify the identified sequence, the deduced amino acid sequence of the neurotoxin was compared to known scorpion toxins (Table 2) [18]. The rChcoh-43 sequence shared 46.97% identity with the sodium channel α-toxin Neurotoxin BmKBTx-like (P84810.1) and 50.00% identity with the sodium channel β-toxin Beta-mammal toxin Css2 (2LJM_A). The toxin Chcoh-43, due to its length and identity, could be associated as a β-toxin. The most closely related toxins originate from *Buthus occitanus tunetanus*, a scorpion species native to Tunisia, Africa, and *Centruroides suffusus suffusus,* from Mexico. Moreover, rChcoh-43 possesses six cysteine residues, which play a crucial role in forming three disulfide bridges, a characteristic structural feature of sodium-channel-modifying scorpion toxins. Additionally, the sequence comprises 70 to 80 highly conserved amino acids (Table 1).

### 2.3. Construction of Vectors and Gene Cloning

The cDNA encoding rChcoh-43 was amplified by PCR and initially inserted into the pCR^®^2.1-TOPO^®^ plasmid (Supplementary Materials, Figures S3–S6, Table S2). The resulting construct was then digested at the BamH1 and Pst1 restriction sites to facilitate subcloning into the pQE30 expression vector, enabling the recombinant production of the scorpion toxin. The oligonucleotides used for vector construction are detailed in Table 3 and Supplementary Materials, Table S3. The pQE30 vector facilitated the expression of recombinant proteins carrying an N-terminal 6His tag, allowing efficient purification via nickel affinity chromatography (Ni-NTA). To minimize any potential impact of the 6His tag on the toxin’s biological activity, a Factor Xa (FXa) cleavage site was introduced between the tag and the mature Chcoh-43 toxin sequence. The expressed recombinant protein, designated rChcoh-43, is presented in Table 1, along with its deduced amino acid sequence and theoretical molecular mass.

### 2.4. Expression System

Shuffle^®^ or Origami^®^ expression strains, different expression media, and incubation conditions were tested for the expression of the recombinant toxin. The best expression conditions found for the toxin are shown in Supplementary Material in Table S4.

### 2.5. Evaluation of Levels of CD95 on the Surface of MCF-7 Breast Cancer Cells

**Figure 1:**
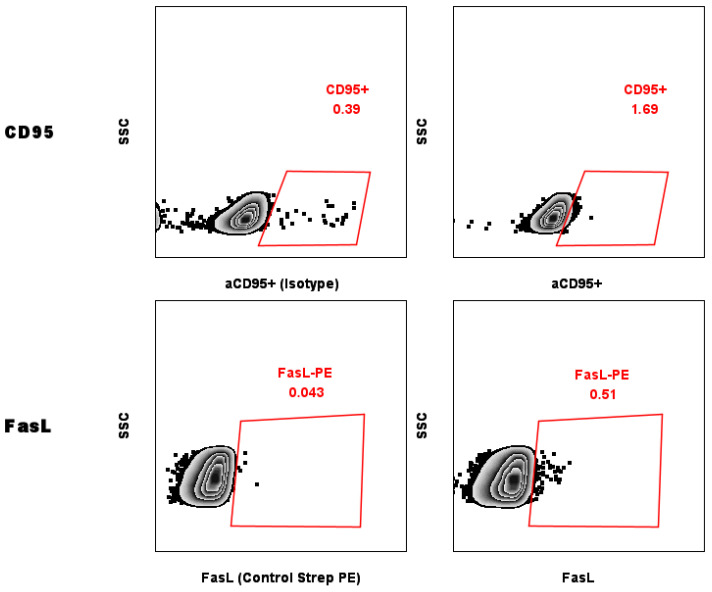
Identification of Fas (CD95) expression by flow cytometry in the MCF-7 cell line using an antiCD95 antibody and FasL-biotin reagent; isotype IgG and streptavidin-PE alone were used as controls. For each experiment 50,000 events were acquired in a Attune NXT flow cytometer, and data were analyzed in FlowJo.

Flow cytometry was used to measure CD95 levels on the surface MCF-7 cells. Culturing cells did not alter the basal level of CD95, regardless of whether an antibody or a ligand (FasL) was used (Figure 1).

### 2.6. Assessment of Cell Death Using 7-AAD by Flow Cytometry: Dose–Response Curve of the Effect at 48 h Doxorubicin Exposure

**Figure 2:**
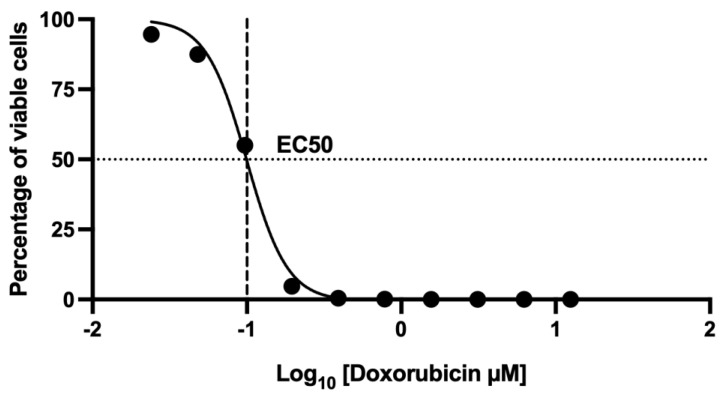
Assessment of viable cells using 7AAD staining by flow cytometry. Dose–response curve illustrating the effect of doxorubicin exposure for 48 h on MCF-7 breast cancer cells. The analysis was conducted using serial dilutions ranging from 12.5 µM to 0.024 µM. Data were acquired via flow cytometry (Attune NxT) to evaluate the impact of doxorubicin on cell viability.

The half-maximal effective concentration (EC_50_) of doxorubicin was determined to be 0.1 µM, which corresponds to the concentration at which 55 ± 1.2% of the MCF-7 cell population died. This value serves as a critical reference point for subsequent cell viability assays, allowing a standardized and physiologically relevant evaluation of doxorubicin-induced cytotoxicity (Figure 2).

### 2.7. Dose–Response Curve of the Effect at 48 h Doxorubicin and Recombinant rChcoh43 Exposure in the MCF-7 Cell Line

**Figure 3:**
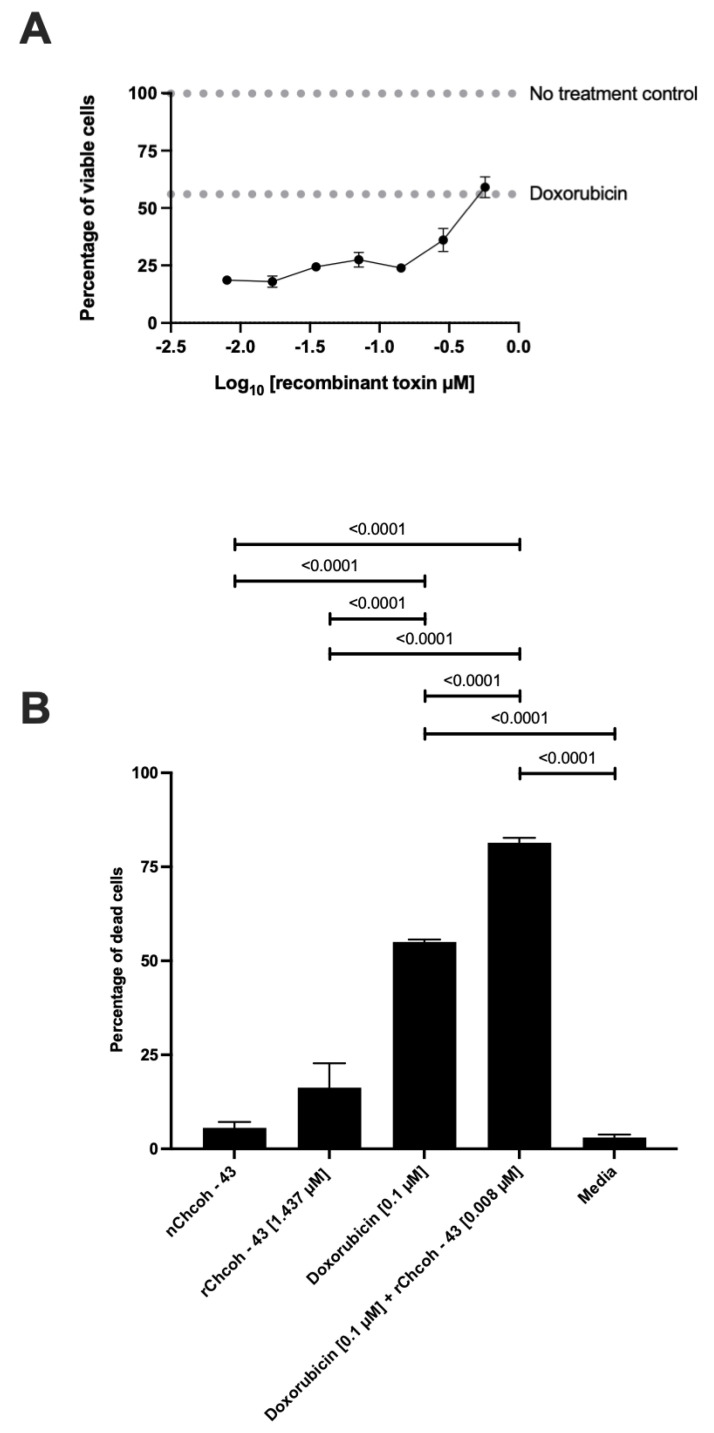
Antagonistic interaction of doxorubicin and recombinant toxin rChcoh43 in MCF-7 breast cancer cells. (**A**) Dose–response curve using doxorubicin at a fixed concentration of 0.1 µM and serial dilutions of rChcoh43 starting at 0.575 µM. (**B**) Comparative analysis of doxorubicin (0.1 µM), rChcoh43 (0.008 µM), and native nChcoh43 toxin, alongside doxorubicin alone, using untreated cells as controls in standard medium. Cell viability was assessed after 48 h of incubation. Data were assessed through an Attune NxT flow cytometer. Experiments were performed in triplicate. Statistical analysis was conducted as described in the Materials and Methods section.

A distinct antagonistic effect was observed at the highest evaluated concentration of rChcoh43 (0.575 µM), where the 55 ± 1.2% of cell death observed with doxorubicin decreased to 40.95 ± 7.85%. As the concentration of rChcoh43 decreased, a progressive reduction in cell viability was detected, with the lowest at 18.6 ± 2.3% at 0.008 µM, contrasting with the behavior of doxorubicin alone, which exhibited its highest cytotoxic effect at the highest concentration tested (Figure 3A). Moreover, neither rChcoh43 nor its native counterpart (nChcoh43) alone had a significant impact on MCF-7 cell viability. However, when rChcoh43 was co-administered with doxorubicin, the highest percentage of cell death was achieved at 81.4 ± 2.3%, surpassing the cytotoxic effect observed with doxorubicin monotherapy (Figure 3B). This suggests a potential synergistic interaction between rChcoh43 and doxorubicin at specific concentration ranges, while also revealing an antagonistic response at higher concentrations.

### 2.8. Evaluation of Ki-67 Expression by Flow Cytometry in the MCF-7 Cell Line After 48 h of Incubation with Native (n) and Recombinant (r) Chcoh43 Toxin and Doxorubicin

**Figure 4:**
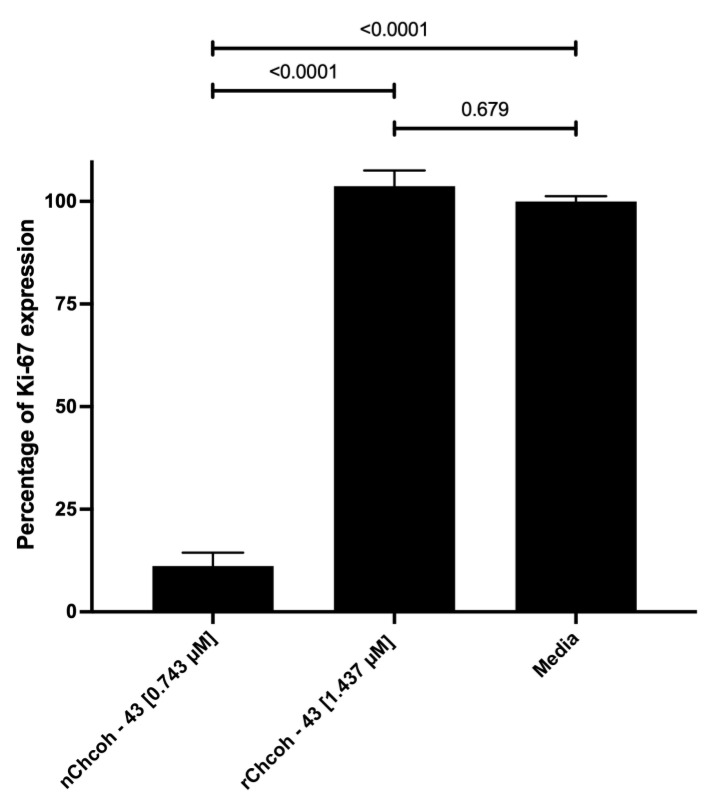
Evaluation of Ki-67 expression in MCF-7 cells by flow cytometry. Cells were incubated for 48 h with native (n) (0.743 µM) and recombinant (r) Chcoh43 (1.437 µM) toxin. Data were acquired by an Attune NxT flow cytometer. Experiments were performed in triplicate. Statistical analysis was conducted as described in the Materials and Methods section.

Cell proliferation was assessed by quantifying Ki-67 expression through flow cytometry. The results indicate that rChcoh43 had no discernible effect on Ki-67 levels, suggesting a lack of influence on cell cycle progression. In contrast, nChcoh43 significantly reduced Ki-67 expression from 100 ± 2.2% to 11.15 ± 5.6%, indicating a potential inhibitory effect on cell proliferation (Figure 4). This differential response suggests structural or functional variations between the native and recombinant forms of the toxin that may influence their biological activity.

## 3. Discussion

Chihuahua, the largest state in Mexico, hosts a remarkable diversity of scorpion species, many of which are endemic to the region. While these species are not considered medically significant to humans, their venoms have been increasingly recognized for their pharmacological potential. A major limitation in studying scorpion venoms is the low yield obtained from individual specimens. The advent of recombinant protein expression techniques has overcome this challenge, enabling large-scale production of specific venom components. The first successful expression of a scorpion neurotoxin in Escherichia coli was reported in 1991 with the production of charybdotoxin from *Leiurus quinquestriatus* [19]. Since then, recombinant scorpion toxins have been widely studied for their potential applications, particularly in cancer research.

Scorpion toxins modulate ion channel activity, particularly sodium (Na+) and potassium (K+) channels, which play critical roles in cellular proliferation, migration, and apoptosis [6]. Some of the most dangerous venom components for mammals are long-chain peptides, which are responsible for the neurotoxic symptoms of scorpion envenomation [20]. These effects are primarily linked to Na+ channel dysfunction, leading to either prolonged activation or inactivation [21].

The upregulation of voltage-gated ion channels, particularly Na^+^ and K^+^ channels, has been linked to tumor progression, increased invasiveness, and metastasis in various cancer types [22]. Some scorpion toxins have demonstrated anticancer effects by inhibiting these channels, thereby increasing apoptosis or reducing cell proliferation and migration. Considering the biochemical characteristics of rChcoh-43, where it has 77 amino acids and six cysteine residues (which form three disulfide bridges), its effects on cellular viability and proliferation have become a relevant aspect to explore. The recombinant toxin could serve as a tool for studying ion channel modulation in cancer cells, potentially offering insights into new therapeutic strategies.

Our finding that nChcoh-43 and rChcoh-43 alone had no significant impact on MCF-7 cell viability aligns with previous research by Driffort et al. [23], who reported that Nav1.5 inhibition using ranolazine at 50 µM reduced sodium currents in MDA-MB-231 cells without affecting cell viability. Aroui et al. [24] evaluated the cytotoxicity of maurocalcine (MCa) at 5 µM, which exhibited no cytotoxic effects. Also, Yang et al. [25] reported that phenytoin at 50 µM did not affect cell viability in both MDA-MB-231 and MCF-7 cells. Importantly, they observed that phenytoin reduced migration and invasion in MDA-MB-231 cells but had no effect on MCF-7 cells, supporting the hypothesis that voltage-gated sodium channels are less prominently expressed in MCF-7 cells. Fraser et al. [26] provided further validation of this interpretation by demonstrating that only MDA-MB-231 cells exhibited inward sodium currents among several breast cancer cell lines tested (MDA-MB-231, MDA-MB-468, MCF-7, and MCF-10A). They confirmed that sodium channel blockade with tetrodotoxin significantly reduced invasion and migration in MDA-MB-231 cells, whereas MCF-7 cells remained unaffected. This correlation between ion channel expression patterns and cellular response to channel-targeting agents helps explain our observation that rChcoh-43 alone showed limited direct effects on MCF-7 cells despite its structural homology to sodium channel toxins.

A particular aspect of our study is the investigation of combined effects between scorpion toxins and conventional chemotherapeutics. Our most notable finding—the bidirectional interaction between rChcoh-43 and doxorubicin—represents a novel observation not previously reported with scorpion toxins. At the highest concentration tested (0.575 µM), rChcoh-43 reduced doxorubicin-induced cell death from 55 ± 1.2% to 40.95 ± 7.85%, exhibiting antagonistic effects. However, at lower concentrations (0.008 µM), it enhanced cytotoxicity to 81.4 ± 2.3%, demonstrating significant synergism. This finding contrasts with the work of Aroui et al. [24], who studied the combination of MCa, a toxin that activates the RyR receptor and functions as a cell-penetrating peptide, with doxorubicin, one of the most widely used chemotherapeutic agents, particularly for breast cancer treatment. Interestingly, they found that an MCa-doxorubicin conjugate reduced drug sensitivity in MCF-7 cells, with the conjugate inducing only 18.6% cell death compared to 34.9% with doxorubicin alone at 5 µM. In contrast, our low-concentration combination achieved substantially higher cytotoxicity than either agent alone. The bidirectional effect we observed might be explained by several molecular mechanisms. At higher concentrations, rChcoh-43 could compete with doxorubicin for cellular uptake or binding sites, activate protective cellular responses, or modulate membrane potential in ways that reduce doxorubicin’s ability to intercalate with DNA. Conversely, at lower concentrations, rChcoh-43 might subtly alter membrane permeability or ion homeostasis, enhancing doxorubicin accumulation within cells or sensitizing them to its cytotoxic effects without triggering compensatory protective mechanisms.

The clinical implications of our findings are significant. Doxorubicin is a cornerstone of breast cancer treatment, but its utility is limited by dose-dependent cardiotoxicity and the development of resistance. Our demonstration that low concentrations of rChcoh-43 can significantly enhance doxorubicin’s cytotoxic effects suggests the potential for developing combination therapies that could reduce the required doxorubicin dosage while maintaining or improving efficacy, potentially mitigating adverse effects and overcoming resistance mechanisms.

CD95 (Fas) receptor triggers the apoptotic cascade through the extrinsic pathway. However, some neoplastic cells develop resistance to apoptosis due to the underexpression of this receptor, contributing to the acquisition of malignant traits. Our finding that this receptor’s expression was absent in MCF-7 cells corroborates previous reports of Fas-mediated apoptosis resistance in this cell line [27]. This resistance to extrinsic apoptosis pathways makes our observation of enhanced doxorubicin-induced cell death particularly noteworthy, as it suggests that the combination therapy may overcome this resistance mechanism, possibly by amplifying intrinsic apoptotic pathways or activating alternative cell death mechanisms.

We also found that nChcoh-43 significantly reduced proliferation by measuring Ki-67 expression to 11.15 ± 5.6% of control levels, in contrast to Yang et al. [25]. This suggests that nChcoh-43 may target additional cellular pathways beyond ion channels. This difference between native and recombinant forms points to the importance of specific structural conformations that might be present in the native toxin but absent in the recombinant version. Potential mechanisms could involve interactions with growth factor receptors, cell cycle regulators, or alternative signaling pathways independent of ion channel modulation. This finding highlights the complexity of scorpion venom components and emphasizes the need for comprehensive characterization of both native and recombinant forms.

The ability of neoplastic cells to invade adjacent tissues, a crucial prerequisite for tumor dissemination and metastasis, has recently been linked to the aberrant expression of voltage-gated sodium (Nav) channels [28]. Driffort et al. demonstrated that silencing Nav1.5 expression prevented pulmonary metastasis in immunosuppressed mice, while treatment with 50 µM ranolazine reduced sodium currents and decreased invasion capacity of MDA-MB-231 cells by 18%. Conversely, 30 µM tetrodotoxin led to a 35% reduction in cellular invasion [23]. Similarly, Yang et al. found that phenytoin reduced migration and invasion in MDA-MB-231 cells [25], an effect that was confirmed by Fraser et al. [26]. While our study does not directly assess invasion and migration, the structural homology of rChcoh-43 to sodium channel toxins suggests potential effects on these processes that merit investigation in future studies.

Our study has several limitations that warrant consideration. We focused primarily on MCF-7 cells, which represent only one molecular subtype of breast cancer. Future research should expand to include additional cell lines representing different molecular subtypes, particularly triple-negative breast cancer, which typically shows poorer outcomes and greater treatment resistance. Additionally, investigating the effects in normal breast epithelial cells would be crucial to assess the specificity of these toxins for cancer cells. In vivo studies would also be necessary to confirm the observed effects in more complex biological systems and to evaluate potential toxicities.

Although *Chihuahuanus coahuilae* is not considered medically important, its venom composition suggests that it may harbor novel bioactive peptides with potential pharmacological potential. The contrasting activities between native and recombinant forms of Chcoh-43 highlight the complexity of scorpion venom components and underscore the need for comprehensive characterization. Further studies on nChcoh-43 and rChcoh-43 will be necessary to determine its specific effects on Na+ and K+ channels, as well as the precise molecular mechanisms relevant to cancer biology. The findings of this study contribute to the growing body of evidence supporting the biomedical relevance of scorpion venom toxins and highlight the importance of characterizing species that, while not posing a direct health risk, may serve as sources of valuable bioactive compounds.

## 4. Conclusions

In conclusion, *Chihuahuanus coahuilae* emerges as a promising source of antineoplastic compounds with potential applications in breast cancer therapy. Our findings demonstrate that the recombinant variant, rChcoh43, when co-administered with doxorubicin at specific concentrations, significantly enhances cell death, suggesting potential for improved therapeutic efficacy through combination treatments. This effect could allow lower doses of conventional chemotherapeutics, potentially reducing adverse effects while maintaining or improving treatment outcomes. Additionally, the native variant, nChcoh43, was found to inhibit cellular proliferation, as evidenced by markedly reduced Ki-67 expression. These findings are particularly relevant as uncontrolled proliferation and apoptosis resistance are key hallmarks of cancer progression. The development of ion-channel-targeting peptides as selective anticancer agents represents an innovative approach that could complement conventional therapies and potentially overcome treatment resistance, ultimately improving outcomes for breast cancer patients. These results underscore the encouraging anticancer properties of venomous animals, providing a foundation for further research into their potential applications in cancer treatment, particularly as a complement to conventional chemotherapies.

## 5. Materials and Methods

### 5.1. Cell Culture and Reagents

MCF-7 cells (ATCC, HTB-22) were cultured in RPMI 1640 media (GIBCO, 11875-085, Paisley, SCT, UK), supplemented with 5% fetal bovine serum (FBS) (GIBCO, 26140-079, Grand Island, NE, USA) and GlutaMAX (GIBCO, 35050-061, Grand Island, NE, USA). The following reagents were used: PBS (SIGMA-ALDRICH, D8537, St. Louis, MO, USA), trypan blue (SIGMA-ALDRICH, T8154, St. Louis, MO, USA), 0.25% trypsin-EDTA (GIBCO, 25200-056, Grand Island, NE, USA), 96-well plates, and 75 cm² cell culture flasks. Flow cytometry staining reagents included anti-Fas(SIGMA-ALDRICH, 05-201, Temecula, CA, USA), FasL-biotin (MBL, D041-6, Woburn, MA, USA), anti-IgG2a isotype control (BioLegend, 401502, San Diego, CA, USA), IgG R-PE (Jackson ImmunoResearch, 115-115-164, West Grove, PA, USA), streptavidin-PE (eBioscience, 12-4317-87, Waltham, MA, USA), and Ki-67 (BioLegend, 350506, San Diego, CA, USA), among others. Doxorubicin (GOLDBIO, D-490-500, St. Louis, MO, USA), Annexin V (BD Pharmingen, 51-65875X, San Diego, CA, USA), 7AAD (BD Pharmingen, 51-68981E, San Diego, CA, USA) were used for cytotoxicity and proliferation assessments. All flow cytometry analyses were performed using the Attune NxT Flow Cytometer.

### 5.2. RNA Extraction from Chihuahuanus Species

Venom glands were obtained from healthy *Chihuahuanus coahuilae* and *Chihuahuanus crassimanus* specimens 48 h after venom extraction. Tissues were immediately preserved in RNAlater™ (Thermo Fisher, Asheville, NC, USA) and stored at −70 °C until further processing. RNA was extracted using the RNeasy Mini Kit (Qiagen Inc., Germantown, MD, USA) and the SV Total RNA Isolation System (Promega Co., Madison, WI, USA). Oligonucleotides were designed based on the N-terminal partial sequence to amplify the corresponding transcript. RNA extraction followed previously described protocols [29].

### 5.3. cDNA Library Construction and Gene Cloning

Isolated RNA was used for cDNA synthesis with an adapter primer (AUAP) 5′(GGCCACGCGTCGACTAGTAC)3′ from the 3′ RACE System for Rapid Amplification of cDNA Ends (Invitrogen, Carlsbad, CA, USA). The thermocycler conditions (Eppendorf, Hamburg, Germany) included an initial denaturation at 94 °C for 3 min, followed by 30 cycles at 94 °C for 1 min, 55 °C for 1 min, and 72 °C for 1 min, with a final extension at 72 °C for 10 min. The transcript was amplified using Taq DNA polymerase (Thermo Fisher Scientific, Waltham, MA, USA). PCR products were purified using the High Pure PCR Product Purification Kit (Roche LifeScience, Penzberg, Germany), ligated into the pCR^®^2.1-TOPO TA cloning vector (Invitrogen, Carlsbad, CA, USA), and transformed into *E. coli* XL1-Blue cells. Clones were analyzed by colony PCR and sequenced at the Instituto de Biotecnología, UNAM, Mexico (additional information on Supplementary Materials, Table S3). The gene encoding toxin 43 was cloned into the pQE30 expression vector and verified by sequencing.

### 5.4. Expression and Purification of the Recombinant Protein

The Shuffle^®^ *E. coli* strain (New England Biolabs^®^ Inc., Ipswich, MA, USA) was transformed with plasmids and grown overnight on LB agar supplemented with 100 μg/mL ampicillin. A single colony was inoculated into LB medium, incubated at 37 °C with shaking, and induced with 1 mM IPTG (Promega, Madison, WI, USA) at OD_600_ 0.6–0.8. The culture was incubated for 16 h at 16 °C, and cells were harvested by centrifugation. The recombinant toxin was purified using Ni-NTA affinity chromatography (QIAGEN, Montgomery, MD, USA) and further purified by RP-HPLC using a C18 column (Supplementary Materials, Figures S4–S6) (Vydac, WR Grace & Co.-Conn, Hesperia, CA, USA). SDS-PAGE confirmed the purification (Supplementary Materials, Figure S7), and the molecular mass was validated by mass spectrometry.

### 5.5. Cell Culture

MCF-7 cells were cultured in RPMI medium with medium changes every two days. Cells were detached with 0.25% trypsin, centrifuged at 2500 RPM for 5 min, and resuspended in medium. Cell density was determined using a Neubauer chamber and trypan blue staining:

Cell Density=(Number of viable cells counted)/(Number of quadrants counted)∗Dilution Factor(2)∗10,000∗Suspension Volume

For experiments, 1 × 10^5^ cells/well were seeded in 96-well plates, incubated at 37 °C with 5% CO_2_, and treated with chemotherapy and Chcoh43 for 48 h. Doxorubicin dose–response curves were generated to determine the EC50, followed by combination assays with toxin Chcoh43.

### 5.6. Detection of Fas/CD95 and FasL/CD178 Expression in MCF-7 Cells

MCF-7 cells (1 × 10^6^) were stained with anti-Fas or anti-IgG2a isotype control, followed by IgG R-PE secondary antibody. FasL expression was detected using anti-Fas-L and streptavidin-PE. Samples were incubated for 30 min at room temperature in darkness and analyzed using the Attune NxT Flow Cytometer.

### 5.7. Cytotoxicity and Proliferation Assays

Cytotoxicity was evaluated using the APC Annexin V/7AAD Kit, where cells were stained, incubated, washed, and analyzed by flow cytometry. For proliferation assays, Ki-67 staining was performed after fixation and permeabilization. Data were analyzed using FlowJo v.X.07. All experiments were conducted in triplicate.

## 6. Statistical Analysis

Dose–response curves of the doxorubicin chemotherapy were analyzed using a variant of the sigmoidal four-parameter log-logistic (4PL) model on the Graph Pad Prism 8.0.1 software to estimate the mean effective concentration (EC50).

$$Y=Bottom+\frac{100-Bottom}{1+10\left(Log\left(AbsoluteIC50\right)-X\right)*HillSlope+log(\frac{100-Bottom}{50-Bottom}-1)}$$

Shapiro–Wilk and Bartlett’s tests were used to evaluate the residuals’ normality and homoscedasticity of the variances (α = 0.05). Significant differences were assessed by analysis of variance (ANOVA and ANOVA max-t+HC3 [30]) between the experimental conditions. All experiments were performed in triplicate, data were expressed as mean ± standard error, and statistical tests were carried out using the R environment version 4.4.1 and Graph Pad Prism 8.0.1.

## Figures and Tables

**Table 1:** Information of the recombinant toxin rChcoh-43.

Toxin	Amino Acid Sequence	No. Amino Acids	MW Theoretic	MW Experiment	No. Cys
rChcoh43	MRGSHHHHHHGSIEGRKKDGYPVNKYGEVSNCVMGMLIGDNTFCKSICRSRGGSGYCYFFACWCEGINDDVKIWKKG**LQ	77	8697.90	8690.31	6

** Stop codon.

**Table 2:** Amino acid sequences of neurotoxins that show identity with the sequence of Chcoh-34.

Database ID	Name	Sequence	(MW) Da
CCD31427.1	Scorpion Toxin To10 precursor	MNYSTLIAVASLLTAGTESKKDGYPVEGSCAFPCGYDNAYCDKLCKERKADSGYCYWVNILCYCYGLPDNAAIKGYGRCKPGKK	9186.54
C9X4K6	Toxin TdNa8	MNYLTLIAAASLLTAGTESKKDGYPVKEGDCAFPCGYDNAYCDKLCKERKADSGYCYWGNILCYCYGLPDKAAIKGYGRCRPGKK	9341
CCD31433.1	scorpion toxin Tpa4 precursor	MNYFVLIAVACLLTAGTESKKDGYPLEYDNCAYDCLGYDNKKCDKLCKDKKADSGYCYWAHILCYCYGLPDNEPIKTSGRCRPGKK	9725.2
P84810.1	Lipolysis-activating peptide 1-alpha chain	MMKLVLFGIIVILFSLIGSIHGISGNYPLNPYGGYYYCTILGENEYCKKICRIHGVRYGYCYDSACWCETLKDEDVSVWNAVKKHCKNPYL	10,457
2LJM_A	Beta-mammal toxin Css2	KEGYLVSKSTGCKYECLKLGDNDYCLRECKQQYGKSSGGYCYAFACWCTHLYEQAVVWPLPNKTCN	7537.6

**Table 3:** Oligonucleotides used for the construction of the expression vector.

Name	Sequence 5-3′
Forward	GAGGATCCATCGAGGGACGCAAAAAGGATGGCTATCCTGTGAAT
Reverse	CTACAGTTCTATACCTTTTTCCCGATTATCGACGTCCTCT

## Data Availability

The original contributions presented in this study are included in the article, supplementary material and the published article (https://doi.org/10.3390/toxins15070416). Further inquiries can be directed to the corresponding author.

## Supplementary Materials

The following supporting information can be downloaded at: https://www.mdpi.com/article/10.3390/toxins17040166/s1, Figure S1. Venom separation analysis of *Chihuahuanus coahuilae*; Figure S2. Repurification of peak 43 from *Chihuahuanus coahuilae* venom was performed using reverse-phase HPLC with a 20–60% acetonitrile gradient containing 0.1% TFA over 45 min on an analytical C18 column; Figure S3. Individual clones were analyzed by colony PCR using a Mastercycler Gradient thermocycler (Eppendorf, Hamburg, Germany) under the following conditions: an initial denaturation at 94 °C for 3 min, followed by 30 cycles at 94 °C for 1 min, 55 °C for 1 min, and 72 °C for 1 min, with a final extension step at 72 °C for 10 min using M13-Forward (5′-GTAAAACGACGGCCAG-3′) and M13-Reverse (5′-CAGGAAACAGCTATGAC-3′) primers; Figure S4. Purification by RP-HPLC using a SUPELCO analytical C18 column with a gradient of 0–60% B in 60 min at a flow rate of 1 mL/min; Figure S5. Purification of the folded protein by RP-HPLC using a gradient of 0–60% B in 60 min on a Vyadac 218TP104 analytical C4 column; Figure S6. Repurification of peak 43 from the folded protein by reverse-phase HPLC, using a 0–60% acetonitrile gradient with 0.1% TFA over 60 min on an analytical C18 column; Figure S7. 12% acrylamide SDS-PAGE gel electrophoresis; Table S1. The toxic effect of different isoforms was tested in mammals (mouse) and insects (cricket); Table S2. The plasmid was extracted from clones 1, 2, 3, 4, 5, and 6 and sent for sequencing. Clones 2 and 3 yielded a positive result; Table S3. Description of the oligonucleotides used to obtain the toxin gene; Table S4. Conditions for the expression of the recombinant toxin rChcoh43.
