# Supplementary material for: Ion-Channel-Targeting Scorpion Recombinant Toxin as Novel Therapeutic Agent for Breast Cancer
Source: Toxins (Basel). 2025 Mar 26;17(4):166. doi: 10.3390/toxins17040166 (PMC12030950; doi:10.3390/toxins17040166)
Supplement: Supplementary file 1 [file toxins-17-00166-s001.zip › toxins-3512214-supplementary.pdf]

# Supplementary Materials: Ion Channel-Targeting Scorpion Recombinant Toxin as Novel Therapeutic Agent for Breast Cancer

## Section 1

The venom was fractionated by reverse phase-HPLC (Agilent 1260 Infinity II, Santa Clara, CA, USA) using an analytical C18 column (Supelco Inc. Sigma-Aldrich, Discovery, 25 cm length and 4.6 mm diameter, 5 m particle size, St. Louis, MO, USA). The column was stored in 0.1% trifluoroacetic acid (TFA). For elution, a gradient was used of acetonitrile (0 to 60%) used as the second solvent 0.1% TFA (Sigma-Aldrich St. Louis, MO, USA). HPLC analysis was for 60 min, mobile phase flow rate 1 mL/min. UV-Vis detector adjusted at 230 nm (Figure 1). The repurification of the fraction 43 was by RP-HPLC (Agilent 1260 Infinity II, Santa Clara, CA, USA) using an analytical C18 column (Supelco Inc. Sigma-Aldrich, Discovery, 25 cm length and 4.6 mm diameter, 5 m particle size, St. Louis, MO, USA). The column was stored in 0.1% trifluoroacetic acid (TFA). For elution, a gradient was used of acetonitrile (20 to 60%) used as the second solvent 0.1% TFA (Sigma-Aldrich St. Louis, MO, USA). HPLC analysis was for 45 min, mobile phase flow rate 1 mL/min (Figure 2).

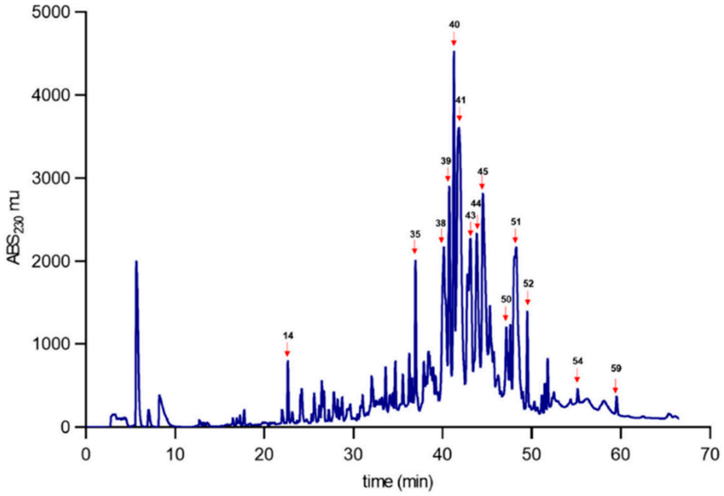


**Figure S1.** Venom separation analysis of Chihuahuanus coahuilae. The venom obtained from scorpionswas separated by RP-HPLC, using a C18 column in a 0 to 60% acetonitrile linear gradient, in 0.1% TFA.Selected fractions marked with numbers and red arrows were further analyzed by mass spectrometry (Alvarado-Gonzalez et al 2023).

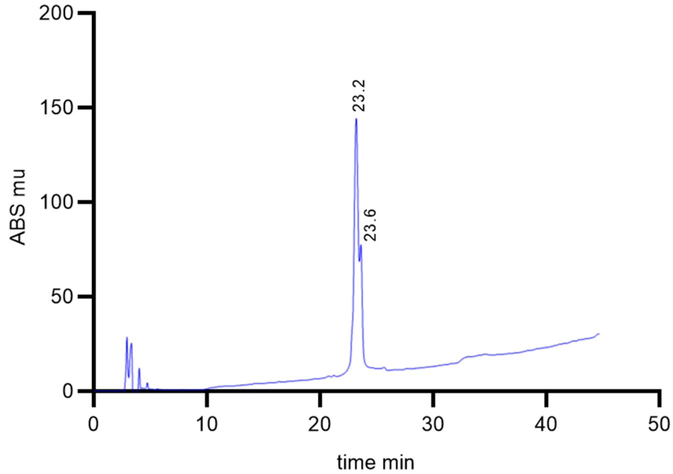


**Figure S2.** Repurification of peak 43 from *Chihuahuanus coahuilae* venom was performed using reverse-phase HPLC with a 20–60% acetonitrile gradient containing 0.1% TFA over 45 minutes on an analytical C18 column. Data were analyzed and plotted using GraphPad Prism software.

## Section 2

**Table S1.** The toxic effect of different isoforms was tested in mammals (mouse) and insects (cricket). The strain of mice used was CD-1, with a weight of 18-20 grams, administered via intracranial injection. Domestic crickets (*Achaeta domesticus*) were injected intrathoracically between the second and third pairs of legs. The following table describes the details.

| | μg | Model | n | Dead | Alive | Observations |
|---|---|---|---|---|---|---|
| 43 | 3 | Mouse | 2 | 0 | 2 | Agitation, piloerection, pain |
| 44 | 3 | Mouse | 2 | 0 | 2 | - |
| 45 | 3 | Mouse | 2 | 0 | 2 | - |
| 43 | 5 | Mouse | 3 | 0 | 3 | Paralysis, agitation, piloerection, pain |
| 44 | 5 | Mouse | 1 | 0 | 1 | - |
| 45 | 5 | Mouse | 1 | 0 | 1 | - |
| 43 | 5 | Cricket | 1 | 0 | 1 | - |
| 44 | 5 | Cricket | 2 | 2 | 0 | Dead after 24 hrs |
| 45 | 5 | Cricket | 2 | 0 | 2 | - |

Colony PCR from the TOPO 2.1 cloning of bands to obtain the sequence of the Chcoh43 toxin.

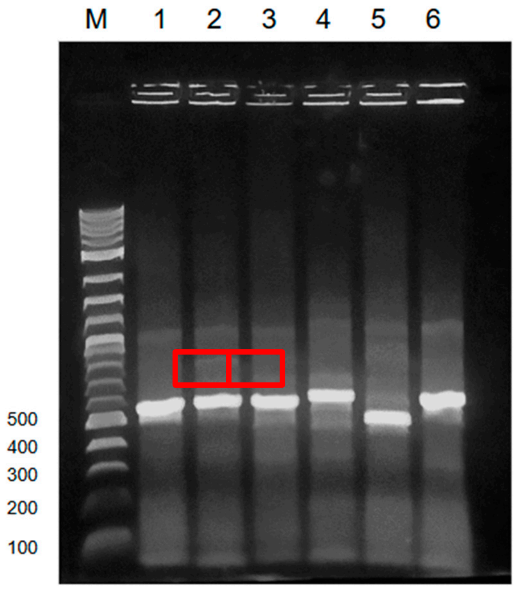


**Figure S3.** Individual clones were analyzed by colony PCR using a Mastercycler Gradient thermocycler (Eppendorf, Hamburg, Germany) under the following conditions: an initial denaturation at 94 °C for 3 min, followed by 30 cycles at 94 °C for 1 min, 55 °C for 1 min, and 72 °C for 1 min, with a final extension step at 72 °C for 10 min using M13-Forward (5′-GTAAAACGACGGCCAG-3′) and M13-Reverse (5′-CAGGAAACAGCTATGAC-3′) primers.

**Table S2.** The plasmid was extracted from clones 1, 2, 3, 4, 5, and 6 and sent for sequencing. Clones 2 and 3 yielded positive results.

| Clone 2 No sec. 11768 | No. aa | MW | Cys (C) |
|---|---|---|---|
| MERTTDTINYTATGSFPYRRIVVYRMKCYAFLTLCLIVLF-FECSDGKKDGYPVNKYG EVSNCVMGMLIGDNTFCKSICRSRGGSGYCYFFACWCEGINDDVKI-WKKG | 61 | 6843.90 | 6 |
| Clone 3 No sec. 11769 | | | |
| MDICRIRPYGRTTDTINYTATGSFPYRRIVVYRMKCYAFLTLCLIVLF-FECSDGKKDG YPVNKYGEVSNCVMGMLIGDNTFCKSICRS-RGGSGYCYFFACWCEGINDDVEIWKKG | 61 | 6844.84 | 6 |

**Table S3.** Description of the oligonucleotides used to obtain the toxin gene.

| Description | Oligonucleotides | Tm | Nt |
|---|---|---|---|
| Forward 10212 | GGA TCC ATC GAG GGA CGC AAA AAG GAT GGC TAT CCT GTG AAT | 56 °C | 42 |
| Reverse 10212 | TCT CCT GCA GCT ATT AGC CCT TTT TCC ATA TCT TGA | 55 °C | 36 |

**Table S4.** Conditions for the expression of the recombinant toxin rChcoh43.

| **Condition** | **Details** |
|---|---|
| Cells | E. coli Shuffle |
| Culture medium | LB (Luria-Bertani) |
| Starter culture | 50 mL LB, 18 h, 37 °C, 100 RPM |
| Culture volume | 1 L LB |
| Induction | 0.5 mM IPTG |
| Expression temperature | 16 °C |
| Expression time | 18 h |
| Cell harvest | Centrifugation |
| Cell lysis | Resuspension and cell disruption using a French press |
| Purification method | His-tag purification using Ni-NTA affinity chromatography |
| Expression validation | SDS-PAGE |

## RP-HPLC purification

Fractions obtained from purifications by Ni-NTA affinity chromatography were pooled to mix the elutions. To 100 µL of this mixture, 10 µM of 1,4-dithiothreitol (DTT) was added, and it was incubated at 55°C for 2 hours, followed by RP-HPLC purification.

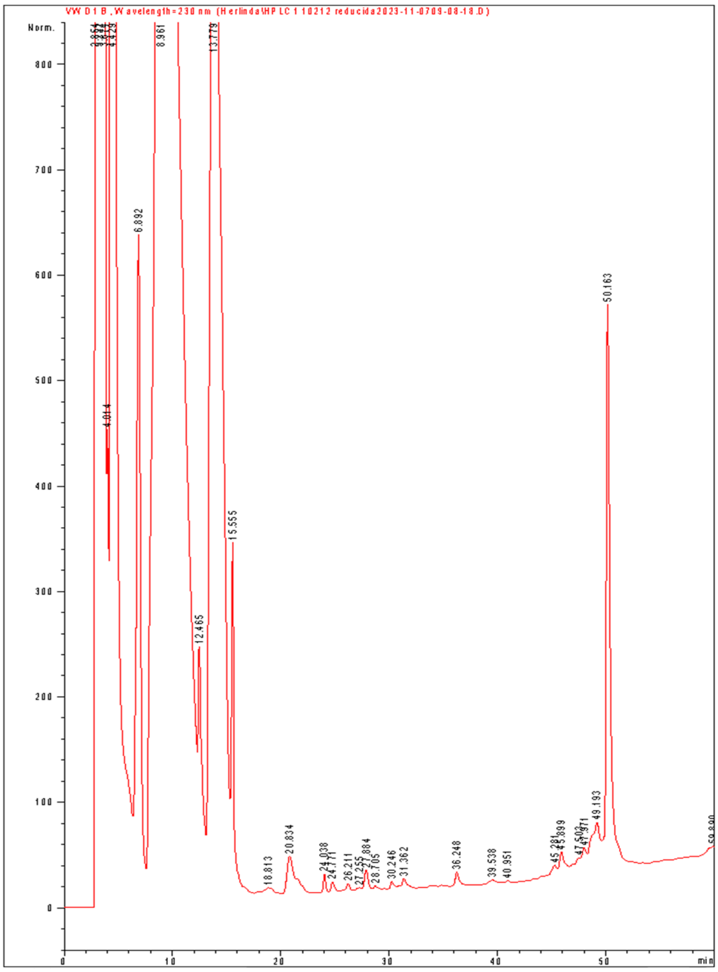


**Figure S4.** Purification by RP-HPLC using a SUPELCO analytical C18 column with a gradient of 0-60% B in 60 minutes at a flow rate of 1 ml/min. The solvents used were: Solvent A: Water (0.1% (v/v) TFA), and Solvent B: Acetonitrile (0.1% (v/v) TFA). Fractions were collected manually, monitored at absorbance of 230 and 280 nm.

## RP-HPLC purification of the reduced protein

200 µg of reduced protein (Cysteine (8M) - Cystine (1M)) was used, agitated for 72 hours at room temperature in a buffer of 50 mM Tris-HCl pH 8, 2M Guanidine Chloride. The Isoforms molecular weight (MW), 43: 8690.31 Da, 44: 8689.08 Da, 45: 8692.2 Da.

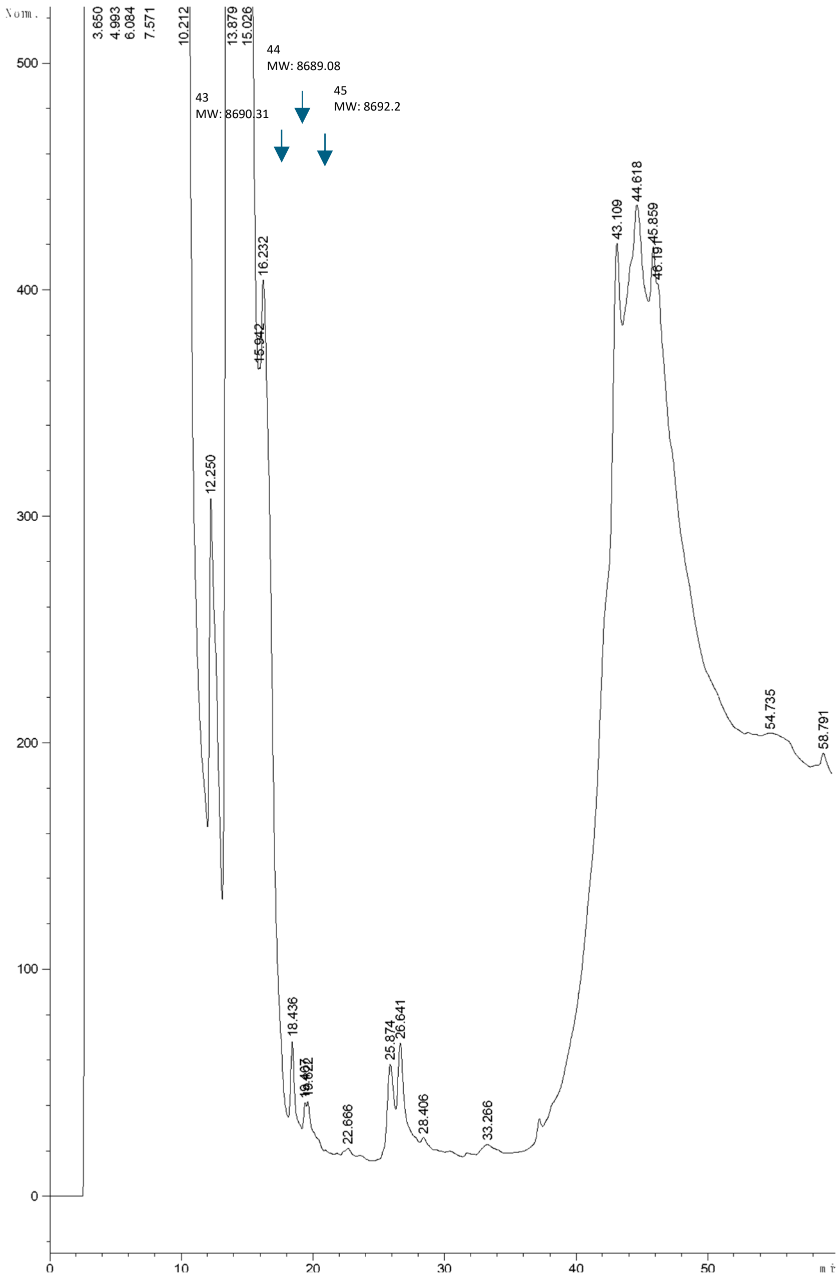


**Figure S5.** Purification of the folded protein by RP-HPLC using a gradient of 0-60% B in 60 min on a Vyadac 218TP104 analytical C4 column. Blue arrows indicate the peaks and their molecular mass. Fractions were collected manually, monitored at absorbance of 230 and 280 nm.

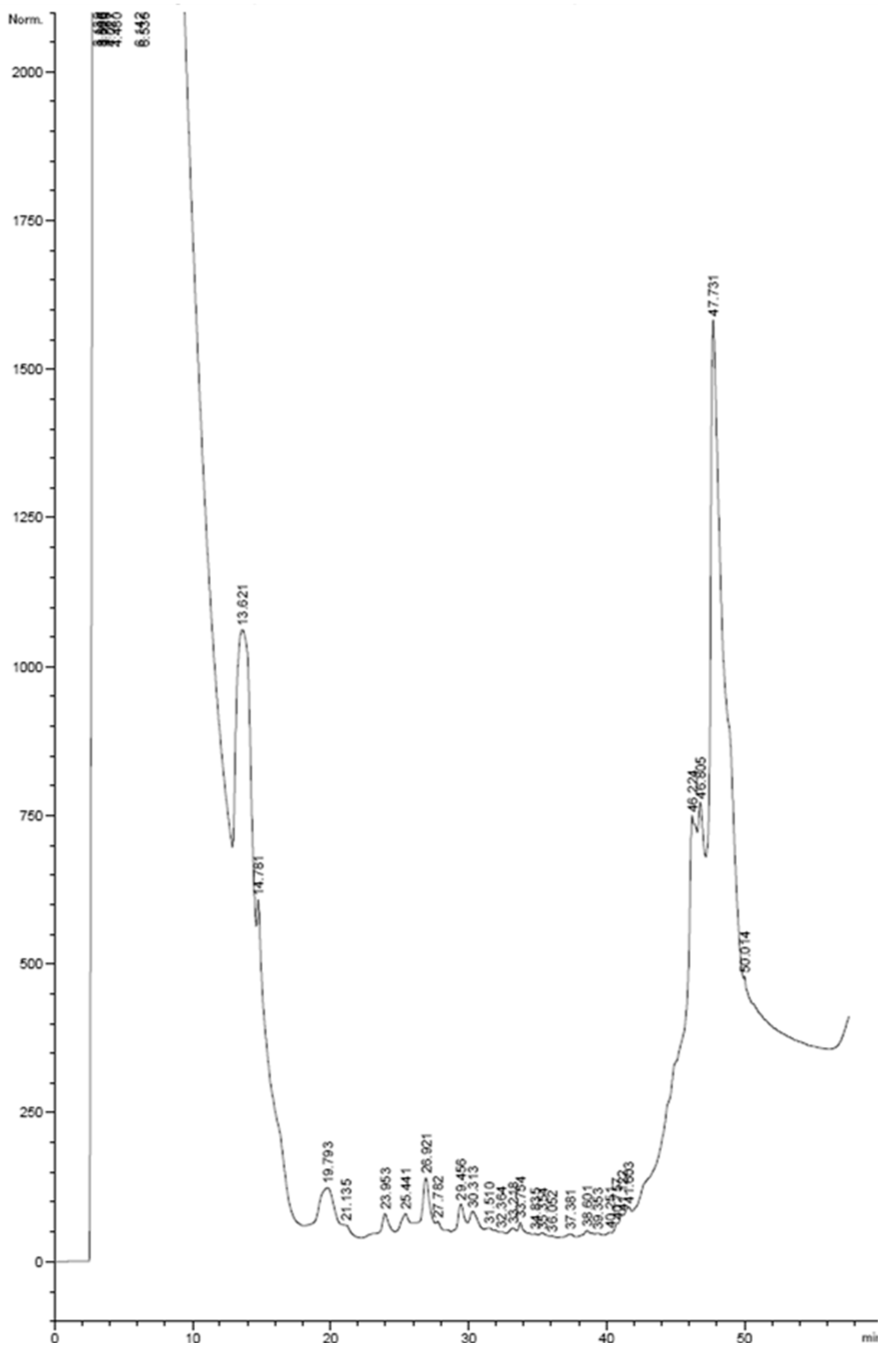


**Figure S6.** Repurification of peak 43 from the folded protein by reverse-phase HPLC, using a 0–60% acetonitrile gradient with 0.1% TFA over 60 minutes on an analytical C18 column. Fractions were collected manually, monitored at absorbance of 230 and 280 nm.

## Expression and Purification of the Toxin by Nickel Affinity Chromatography

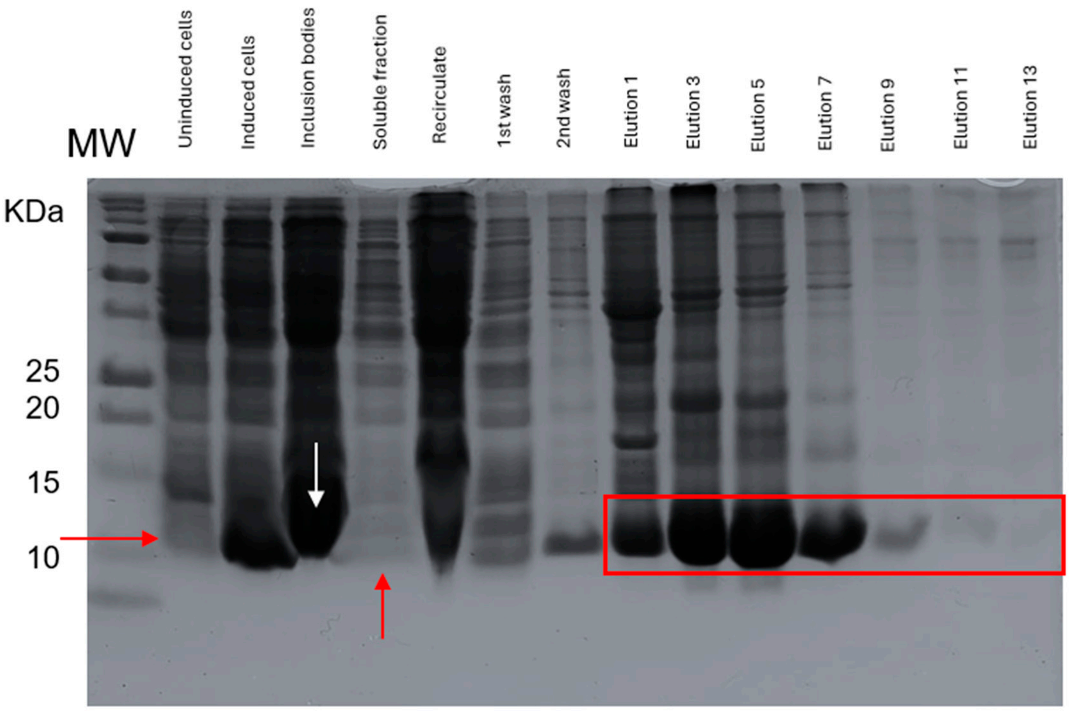


**Figure S7.** 12% acrylamide SDS-PAGE gel electrophoresis. Wells were loaded with: MW, molecular weight marker, 1, uninduced cells; 2, induced cells; 3, inclusion bodies; 4, soluble fraction; 5, recirculate; 6, first wash; 7, second wash; 8-14, elutions of the toxin purified by Nickel Affinity Chromatography.
